# The Use of Chlorophyll Meters to Assess Crop N Status and Derivation of Sufficiency Values for Sweet Pepper

**DOI:** 10.3390/s19132949

**Published:** 2019-07-04

**Authors:** Romina de Souza, M. Teresa Peña-Fleitas, Rodney B. Thompson, Marisa Gallardo, Rafael Grasso, Francisco M. Padilla

**Affiliations:** 1 Department of Agronomy, University of Almeria, Carretera de Sacramento s/n, 04120 La Cañada de San Urbano, Almería, Spain; 2 CIAIMBITAL Research Centre for Mediterranean Intensive Agrosystems and Agrifood Biotechnology, University of Almeria, 04120 La Cañada de San Urbano, Almería, Spain; 3 Estación Experimental INIA Salto Grande, Instituto Nacional de Investigación Agropecuaria (INIA), Camino al Terrible s/n, 50000 Salto, Uruguay

**Keywords:** atLEAF, CCI, greenhouse, horticulture, nitrogen nutrition index, proximal optical sensors, SPAD, vegetable crops

## Abstract

Chlorophyll meters are promising tools for improving the nitrogen (N) management of vegetable crops. To facilitate on-farm use of these meters, sufficiency values that identify deficient and sufficient crop N status are required. This work evaluated the ability of three chlorophyll meters (SPAD-502, atLEAF+, and MC-100) to assess crop N status in sweet pepper. It also determined sufficiency values for optimal N nutrition for each meter for pepper. The experimental work was conducted in a greenhouse, in Almería, Spain, very similar to those used for commercial production, in three different crops grown with fertigation. In each crop, there were five treatments of different N concentration in the nutrient solution, applied in each irrigation, ranging from a very deficient to very excessive N supply. In general, chlorophyll meter measurements were strongly related to crop N status in all phenological stages of the three crops, indicating that these measurements are good indicators of the crop N status of pepper. Sufficiency values determined for each meter for the four major phenological stages were consistent between the three crops. This demonstrated the potential for using these meters with sufficiency values to improve the N management of commercial sweet pepper crops.

## 1. Introduction

To optimize nitrogen (N) fertilizer application, it is necessary to match the N supply to the N demand [1,2]. A potentially very effective approach would be the rapid and frequent on-farm assessment of crop N status that permits rapid adjustment of the N supply [3,4,5]. Proximal optical sensors are a broad group of non-destructive monitoring tools that can be used to assess crop N status [5,6,7]. One particularly promising group of proximal optical sensors are leaf chlorophyll meters.

Chlorophyll meters are relatively simple proximal optical sensors that indirectly assess relative leaf chlorophyll content by measuring the differential absorbance and transmittance of different radiation wavelengths by the leaf [3,7,8]. Given that leaf chlorophyll content is usually related to crop N content [6,9,10], these measurements can be used to assess crop N status [3,7]. Three commercially-available meters, with different characteristics, such as the wavelengths used, are the SPAD-502 meter (Konica-Minolta, Tokyo, Japan), atLEAF+ sensor (FT Green LLC, Wilmington, DE, USA), and MC-100 chlorophyll meter (Apogee Instruments, Inc., Logan, UT, USA) [5,7,11]. The SPAD-502 measures absorbance at 650 nm (red) and 940 nm (NIR), the atLEAF+ at 660 nm and 940 nm, and the MC-100 at 653 nm and 931 nm. Using the two absorbance values, these three meters calculate a dimensionless numerical value, which is related to chlorophyll content [11]. There are also differences in price between these meters; the atLEAF+ sensor is almost 10 times cheaper than the SPAD-502 and the MC-100 meters. The major practical advantages of chlorophyll meters as indicators of crop N status are that they are easy to use, do not require any particular training, and they make measurements very rapidly, with no or very little data processing [3,4,7,12].

Chlorophyll meter measurements do not directly indicate crop N status, so interpretation is required [7]. Two broad approaches have been proposed to interpret chlorophyll meter measurements to assess crop N status. One approach is the use of so-called “reference plots” [13,14]. This approach divides the measured values of the crop by those from a well-fertilized reference plot that has no N limitation [15]. This is considered to isolate the effect of relative N status from other confounding factors that are common to both areas [16], which could greatly facilitate the adoption of chlorophyll meters on farms. However, this approach is considered to be impractical for commercial fertigated vegetable crops, given: (1) the additional cost of having separate fertigation sectors for reference plots, and (2) the implicit assumption of sensor saturation may not apply when luxury N uptake occurs, as has been reported for some vegetable species [5,11].

Another approach to interpret chlorophyll meter measurements, for the assessment of crop N status, is the use of absolute sufficiency values based on direct measurement. The sufficiency value is an absolute value, below which the crop is deficient and responds to additional N fertilizer [3,17], and above which yield is not affected [3] and the immediate N supply may be excessive [5]. Sufficiency values provide information on whether adjustments in N fertilization are required when absolute measurements deviate from sufficiency values [18].

To determine sufficiency values, the nitrogen nutrition index (*NNI*) can be used [7,14]. The *NNI* is an effective and established indicator of crop N status [19] that relates the actual crop N content to the critical crop N content (i.e., the minimum N content necessary to achieve maximum growth of a crop) [20]. Values of *NNI* = 1 correspond to optimal N nutrition [19]. Sufficiency values of chlorophyll meter measurements are derived from the relationship between crop *NNI* and chlorophyll meter measurements by solving the relationship for *NNI* = 1 [7,17]. Chlorophyll meter sufficiency values have been determined for fresh tomato [4,21], and cucumber [17,22]. Sufficiency values are not available for most vegetable species, including important crops such as sweet pepper. Additionally, sufficiency values should be determined for specific agricultural systems and regions.

In Southeast (SE) Spain, the greenhouse-based intensive vegetable production system consists of approximately 40,000 ha of relatively simple plastic greenhouses, most of which are concentrated in the province of Almeria [23,24]. Nitrate (NO_3_^−^) leaching from this system [25] is associated with considerable aquifer NO_3_^−^ contamination [26]. Frequent monitoring of these fertigated vegetable crops with chlorophyll meters is a promising approach to optimize crop N management, which would reduce N fertilizer use, thereby contributing to reduced aquifer NO_3_^−^ contamination. In Almeria, sweet pepper is either the most or second most important crop, depending on the year, occupying approximately 8000 ha each year [27]. Globally, sweet pepper is grown on 1.9 million hectares [28].

The objectives of the present work were: (i) to evaluate the sensitivity of three different chlorophyll meters to assess the crop N status of sweet pepper crops, and (ii) to calculate sufficiency values for each chlorophyll meter for maximum crop growth for four different phenological stages. This work was conducted in three different sweet pepper crops grown in different cropping years (2014–2015, 2016–2017, and 2017–2018) in a greenhouse. In each crop there was five different N treatments, ranging from very deficient to very excessive.

## 2. Materials and Methods

### 2.1. Experimental Site

Three sweet pepper (*Capsicum annuum,* cultivar ‘Melchor’) crops were grown in soil in a plastic greenhouse, in conditions similar to commercial greenhouse vegetable production in SE Spain [27]. The experimental work was conducted at the Experimental Station of the University of Almeria (36°51’N, 2°16’W and 92 m elevation). The greenhouse had polycarbonate walls and a roof of low-density polyethylene (LDPE) tri-laminated film (200 μm thickness), with transmittance to photosynthetically active radiation (PAR) of approximately 60%. It had no heating or artificial light, had passive ventilation (lateral side panels and flap roof windows), and an east–west orientation, with crop rows aligned north–south. The cropping area was 1300 m^2^. The crops were grown in an “enarenado” soil, typical of those used for soil-grown greenhouse production in Almería [25]. A more detailed description of the soil used is available in Padilla et al. [29].

Above-ground drip irrigation was used for combined irrigation and mineral fertilizer application. Drip tape was arranged in paired lines, with 0.8 m spacing between lines within each pair of lines, 1.2 m spacing between adjacent pairs of lines, and 0.5 m spacing between drip emitters within drip lines, giving an emitter density of two emitters m^−2^. The greenhouse was organized into 24 plots, measuring 6 m × 6 m; 20 plots were used in the current study. There were five N treatments with four replicate plots per treatment, arranged in a randomized block design. Each plot contained three paired lines of plants (six lines of plants in total), with 12 plants in each line, separated by a 0.5 m spacing. One plant was positioned 60 mm from and immediately adjacent to each dripper, giving a plant density of two plants m^−2^ and 72 plants per replicate plot. Sheets of polyethylene film (250 µm thickness) buried up to 30 cm depth acted as a hydraulic barrier between plots [30].

### 2.2. Experimental Design

The three sweet pepper crops were grown in different years. The first crop, in 2014–2015 (“the 2014 crop”) was transplanted on 12 August 2014 and ended on 29 January 2015 (cropping period of 170 days). The second crop, “the 2016 crop”, was transplanted on 19 July 2016 and ended on 24 March 2017 (cropping period of 248 days). The third crop, “the 2017 crop”, was transplanted on 21 July 2017 and ended on 20 February 2018 (cropping period of 214 days). The three crops were transplanted as 35-day old seedlings using the same cultivar.

In each crop, there were five treatments of different N concentration in the nutrient solution, applied by fertigation throughout the crops. In the 2014 crop, the N treatments commenced one day after transplanting (DAT), in the 2016 crop at nine DAT, and in the 2017 crop at 10 DAT. Plants were irrigated with water only (<0.04 mmol N L^−1^) prior to commencing the N treatments. The N treatments were applied in every irrigation until the end of the crops. In each crop, the N treatments were very deficient (N1), deficient (N2), conventional (N3), excessive (N4), and very excessive (N5). The average mineral N (NO_3_^−^–N + NH_4_^+^–N) concentrations (mmol L^−1^), applied in the nutrient solution, and the amounts (kg ha^−1^) of N applied in each N treatment in each crop are presented in Table 1. For all treatments, N was applied mostly as nitrate (NO_3_^−^), the rest as ammonium (NH_4_^+^); on average 88% of the N was applied as NO_3_^−^. All other nutrients were applied in the nutrient solution to ensure they were not limiting.

The crops were managed following local practice. The crops were physically supported using a system of nylon cords placed horizontally along the side of the crop. Irrigation was scheduled to maintain soil matric potential (SMP) in the root zone, at 12 cm deep, within −15 to −25 kPa; one tensiometer (Irrometer, Co., Riverside, CA, USA) per plot was used to measure SMP. High temperature within the greenhouse was controlled by white-washing the plastic cladding with CaCO_3_ suspension.

### 2.3. Chlorophyll Meter Measurements

Chlorophyll meter measurements commenced on 27 (15 DAT), 18 (25 DAT), and 11 (21 DAT) August for the 2014, 2016, and 2017 crops, respectively. In the 2014 crop, measurements were made every seven days, and in the 2016 and 2017 crops every 14 days. In the three crops, measurements were made until the end of the crop. Three different leaf-clip chlorophyll meters were used, the SPAD-502 meter (Konica Minolta, Inc., Tokyo, Japan), the atLEAF+ meter (FT Green LLC, Wilmington, DE, USA), and the MC-100 Chlorophyll Concentration Meter (Apogee Instruments, Inc., Logan, UT, USA). The respective measurement values are SPAD units, atLEAF units, and chlorophyll content index (CCI). The SPAD-502 meter was used in each of the three crops (2014, 2016, and 2017). The atLEAF+ meter was used in the 2016 and 2017 crops. The MC-100 meter was used only in the 2017 crop. The areas measured in each measurement were 6 mm^2^ for the SPAD-502, 13 mm^2^ for the atLEAF+, and 63.6 mm^2^ for the MC-100.

Measurements were made on one leaf of each of the 16 marked plants in each replicate plot. The value for each replicate plot was the mean of the 16 individual measurements. They were made at the same time of day (8:00 to 10:00 solar time), before irrigation/fertigation. Measurements were made on each plant on the most recently fully expanded and well-lit leaf, on the distal part of the adaxial side of the leaf, midway between the margin and the mid-rib of the leaf. Measurement was made by clipping the sensor onto the leaf. Leaves with physical damage or with condensed water were not measured, alternative plants being selected.

### 2.4. Determination of Crop Nitrogen Nutrition Index

The critical N curve derived for greenhouse-grown sweet pepper, *Nc* = 4.488·DMP^−0.196^ (A. Rodríguez and R.B. Thompson, University of Almeria, personal communication), where DMP is dry matter production, was used to calculate the nitrogen nutrition index (*NNI*) as a measure of crop N status.

The *NNI* was calculated as:

(1) $$NNI=\frac{Nact}{Nc},$$

where *Nact* is the measured N content of the crop and *Nc* is the critical N content obtained from the critical N curve for each treatment for each biomass sampling date. *NNI* values for each day of chlorophyll meter measurement were calculated by interpolating DMP and crop N content values between the two biomass samplings on either side of the measurement date. Above-ground dry matter production during the crop was measured by periodic biomass sampling (approximately every 14 days) by removing two complete plants in each replicate plot. All fresh material of each biomass component (stem, leaf, and fruit) was weighed, and the dry matter contents determined by oven-drying representative sub-samples at 65 °C until a constant weight was reached. Fruit production and pruning was determined throughout the crop, in eight selected plants in each replicate plot. Representative samples of leaves, stems, and fruit from each biomass sampling, from each replicate plot, were ground sequentially in knife and ball mills. The total N content (%N) of each sample was determined using a Dumas-type elemental analyzer system (model Rapid N, Elementar, Analysensysteme GmbH, Hanau, Germany). The mass of N in each relevant component was calculated from the %N and the corresponding mass of dry matter. Total crop N uptake in each replicate plot, at each biomass sampling, was the sum of N in all relevant components. Crop N content (%N) for each biomass sampling was calculated, for each replicate plot, as crop N uptake divided by DMP.

### 2.5. Data Analysis

To account for differences in planting dates that occur in vegetable crops, and to facilitate the use and interpretation of chlorophyll meters in practical farming, measurements and analyses were based on phenological stage rather than on days after transplanting. Because of the frequent measurements with chlorophyll meters during the pepper cycle, there were several dates of measurements within each phenological stage. To integrate the various dates of measurement to provide a unique value for each phenological stage, integrated values of each chlorophyll meter measurement (*SPADi*, *atLEAFi*, and *CCIi*) and for the crop nitrogen nutrition index (*NNIi*), were calculated for each phenological stage. These integrated values were calculated as:

*Integrated value* = 1/*D* × ∑(*V* × d*s*), (2)

where *D* was the total number of days of each phenological stage, *V* was the value measured at each measurement date, and ds was the interval between two successive measurement dates (values of each measurement date were pondered by the time elapsed between two consecutive measurements). Four major phenological stages were considered: (i) vegetative, (ii) flowering, (iii) early fruit growth, and (iv) harvest. The vegetative stage was from transplanting to the beginning of flowering. The flowering stage was from the beginning of flowering until fruit set. Early fruit growth was from fruit set until fruit maturation. The harvest stage commenced with the first fruit harvest and ended when the crop finished, this was the longest of the four phenological stages.

To evaluate the sensitivity of *SPADi*, *atLEAFi*, and *CCIi*, to estimate crop *NNIi*, regression analyses were performed for each phenological stage. Four types of regression equations (linear, quadratic, power, and exponential) were considered, and the best equation was selected using the Akaike information criterion [31], which represents the best compromise between the goodness of fit and the complexity of a model. These regression analyses were performed for each phenological stage in each crop, and for each crop in its entirety. Additionally, where there was more than one crop in which a particular chlorophyll meter was used (SPAD, atLEAF+), these regression analyses were conducted on: (a) pooled data for each phenological stage from the different crops, and (b) composite whole crop data from the different crops. The CurveExpert Professional® 2.2.0 software (Daniel G. Hyams) was used for these regression analyses.

Sufficiency values of chlorophyll meter measurements for maximum crop growth were derived for each phenological stage from the relationship between integrated chlorophyll meter measurements and *NNIi*. The approach of Padilla et al. [4] was used, in which the Akaike Information Criterion (AIC) best-fit equations that related chlorophyll meter measurements to *NNI* were solved for *NNI* = 1, which is the value of *NNI* that represents the optimal N nutrition for maximum growth. Sufficiency values of chlorophyll meter measurements were calculated for: (a) each phenological stage for each crop considered separately, (b) each whole crop, (c) each phenological stage for multiple crops, and (d) the whole crop using data from multiple crops.

## 3. Results

### 3.1. Effects of N Treatments on the Nitrogen Nutrition Index

**Figure 1:**
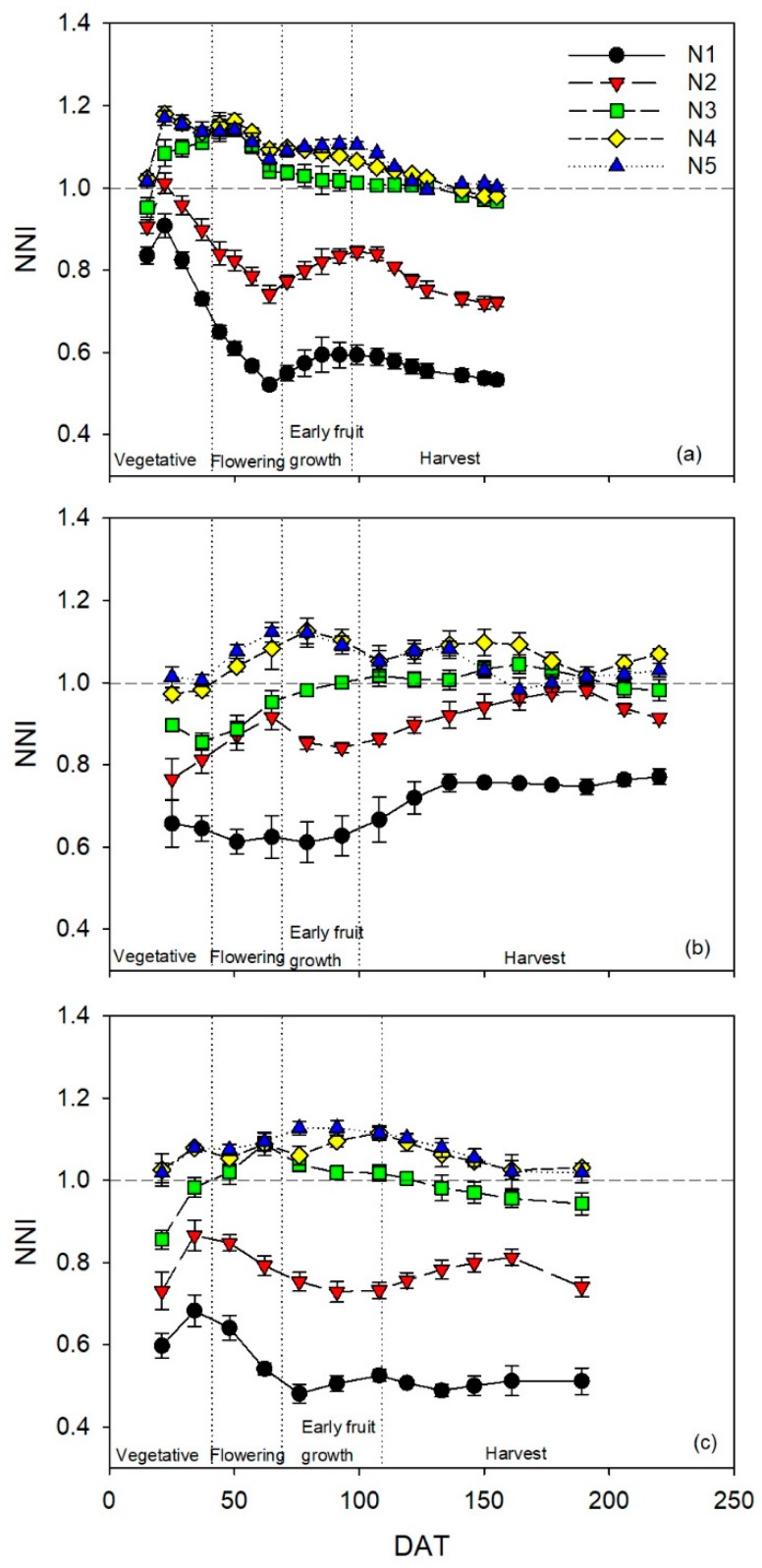
Temporal dynamics of the nitrogen nutrition index (*NNI*) for the sweet pepper (*Capsicum annuum*) crops in the (**a**) 2014, (**b**) 2016, and (**c**) 2017 crops, subjected to five different N treatments with four repetitions. Values are means (*n* = 4) ± standard error (±SE). DAT is days after transplanting. Vertical dotted lines represent the different phenological stages; the horizontal dotted line indicates *NNI* = 1.

In general, throughout the three crops (the 2014, 2016, and 2017 crops), *NNI* was consistently clearly less than one in the N1 and N2 treatments (Figure 1). The exception was the 2016 crop, where the N2 treatment had *NNI* values close to one in the second half of the crop (Figure 1b). In the three crops evaluated, N4 and N5 treatments had values higher than one for most of the crop. The N3 treatment in the three crops had *NNI* values that were consistently close to one for most of the crop (Figure 1).

In each of the three crops, there were significant differences in integrated *NNI* (*NNIi*) values between the N1, N2, N3, and N4 treatments in the vegetative phenological stage (Table 2). There were no significant differences in *NNIi* between the N4 and N5 treatments in the vegetative stage in the three crops (Table 2). Generally, the statistical results from comparing the *NNIi* values of the N treatments were very similar for the flowering, early fruit growth, and harvest phenological stages, in each of the three crops (Table 2). There were some exceptions, mostly when the *NNIi* values of the N3 treatment were not significantly different to those of the N4 treatment (Table 2).

### 3.2. Effects of N Treatments on Chlorophyll Meters Measurements

**Figure 2:**
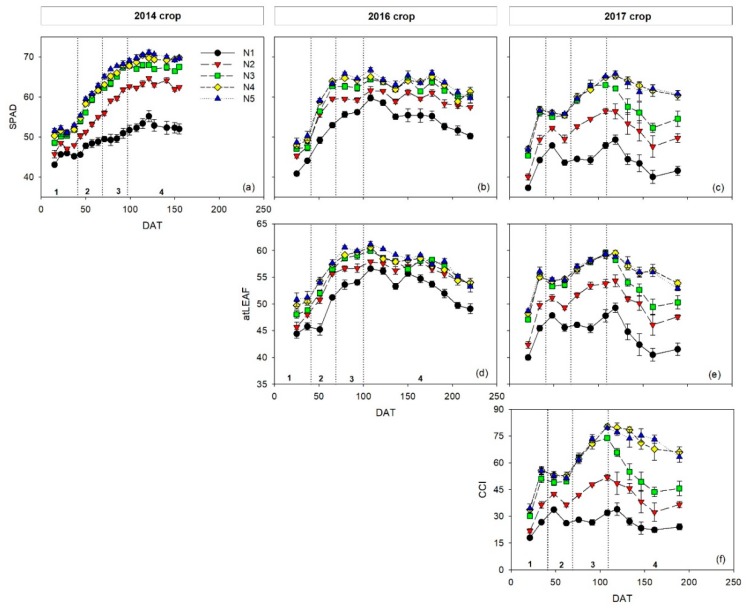
Temporal dynamics of chlorophyll meters measurements of SPAD values (**a**–**c**), atLEAF values (**d**,**e**), and CCI values (**f**), for the sweet pepper (*Capsicum annuum*) crops subjected to five different N treatments with four repetitions. Vertical dotted lines and numbers represent the different phenological stages: 1—vegetative, 2—flowering, 3—early fruit growth, 4—harvest. Values are means (n = 4) ± standard error (SE). DAT is days after transplanting.

The temporal dynamics of measurements with the three chlorophyll meters (SPAD-502, atLEAF+, and MC-100) throughout the crops were very similar, regardless of the chlorophyll meter (Figure 2). Generally, treatment N1 had the lowest values, treatment N2 was lower than treatments N3, N4, and N5, treatments N4 and N5 were the highest and were very similar, and treatment N3 was often intermediate between treatments N2 and N4. At times, values from treatments N3, N4, and N5 were all similar.

In each of the three crops (2014, 2016, and 2017) there were generally significant differences in integrated SPAD (*SPADi*) values between the N1, N2, and N3 treatments in each phenological stage (vegetative, flowering, early fruit growth, and harvest) (Table 3). There were generally no significant differences in *SPADi* values between the N3 and N4 treatments. In all of the phenological stages of crops, there were no significant differences in *SPADi* between the N4 and N5 treatments (Table 3).

Regarding the atLEAF+ meter, there were significant differences in integrated atLEAF (*atLEAFi*) values between the N1 and N2 treatments in all phenological stages in the 2016 and 2017 crops (Table 4). However, for the 2016 crop in most phenological stages there were no significant differences in *atLEAFi* values between the N2 and N3, but there were significant differences between these treatments in the 2017 crop. For both crops there were no significant differences between the N3 and N4 treatments, and between the N4 and N5 treatments in most phenological stages (Table 4).

For integrated CCI values (*CCIi*) measured with the MC-100 meter in the 2017 crop, there were significant differences in *CCIi* between N1 and N2 treatments, and between N2 and N3 treatments (Table 5). There were no significant differences in *CCIi* between the N4 and N5 treatments (Table 5).

### 3.3. Relationships between Integrated Chlorophyll Meters Measurements and Integrated NNI

**Figure 3:**
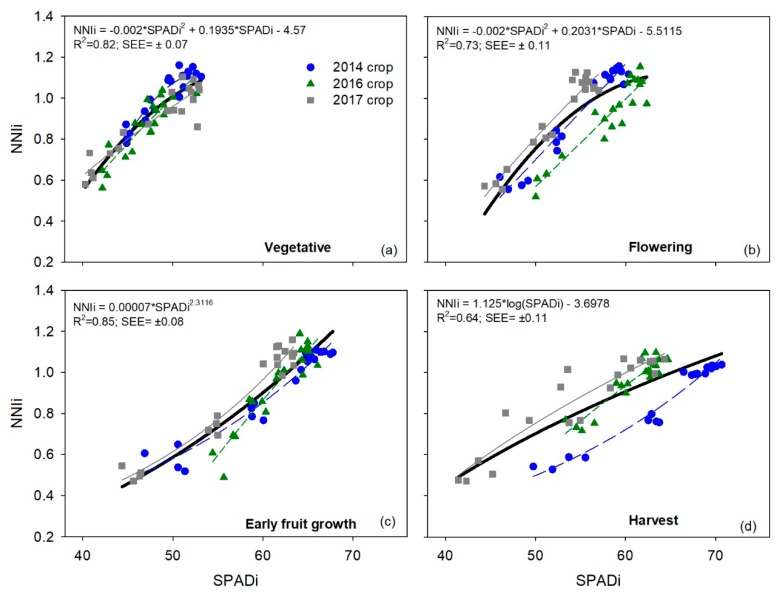
Relationships between integrated SPAD (*SPADi*) values and the integrated crop nitrogen nutrition index (*NNIi*) for each phenological stage in each of the three sweet pepper (*Capsicum annuum*) crops. Each crop was subjected to five different N treatments with four repetitions. The bold black line and the equation represent the adjustment for the combined dataset of the three crops together. Results of regression for each crop separately are in Table 6.

Relationships between *SPADi* and *NNIi* values for each phenological stage in each of the three crops had coefficients of determination (*R*^2^) of 0.89–0.97, 0.80–0.88, and 0.86–0.96 for the 2014, 2016, and 2017 crops, respectively (Table 6). When averaged for the duration of each crop, the *R*^2^ values of the 2014, 2016, and 2017 crops were 0.93, 0.86, and 0.90, respectively (Table 6). Generally, in each of the three crops, the relationships between *SPADi* and *NNIi* values had similar R^2^ values for the different phenological stages (Table 6). Combining the three crops together, the R^2^ values of the four phenological stages ranged from 0.64 (harvest) to 0.85 (early fruit growth), with an average R^2^ value of 0.76 across the four phenological stages (Table 6). There was no evidence of saturation of SPAD values at higher *NNI* values in any of the four phenological stages in any of the three crops (Figure 3). Regression analysis showed that SPAD values increased when *NNI* values exceeded the optimal value, for instance, for crop growth.

**Figure 4:**
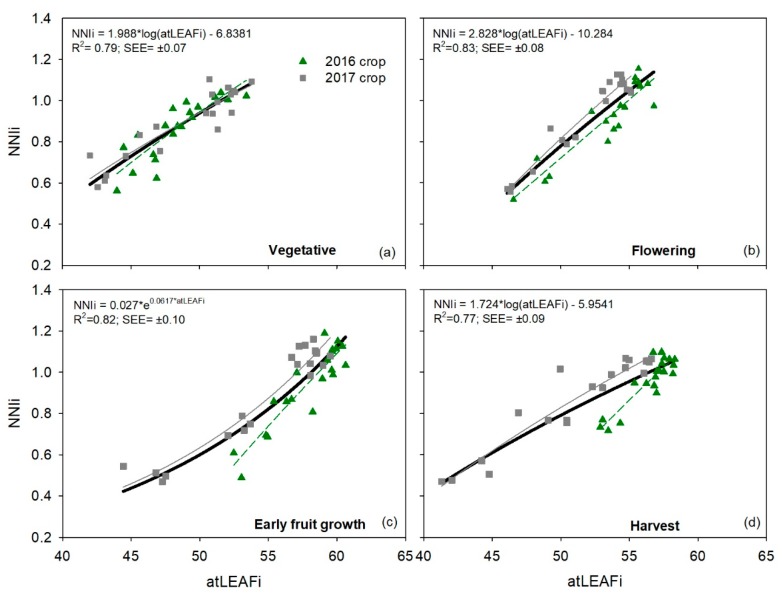
Relationships between integrated atLEAF (*atLEAFi*) values and the integrated crop nitrogen nutrition index (*NNIi*) for each phenological stage in each of the two sweet pepper (*Capsicum annuum*) crops. Each crop was subjected to five different N treatments with four repetitions. The bold black line and the equation represent the adjustment for the combined dataset of the two crops together. Results of regression for each crop separately are in Table 7.

For the atLEAF+ meter, relationships between *atLEAFi* values and *NNIi* values for each phenological stage, in each of the crops, had R^2^ values of 0.74 to 0.94 (Table 7). Averaged for each crop, *R*^2^ values were 0.81 and 0.90 for the 2016 and 2017 crops, respectively (Table 7). There were no appreciable differences between the four phenological stages within each crop (Table 7). Combining the data of the two crops, the R^2^ values of the four phenological stages ranged from 0.77 (harvest) to 0.83 (flowering), with an average R^2^ value for the two entire crops of 0.80 (Table 7). There was no appreciable saturation of atLEAF values at higher *NNI* values in any of the four phenological stages in the two crops (Figure 4).

**Figure 5:**
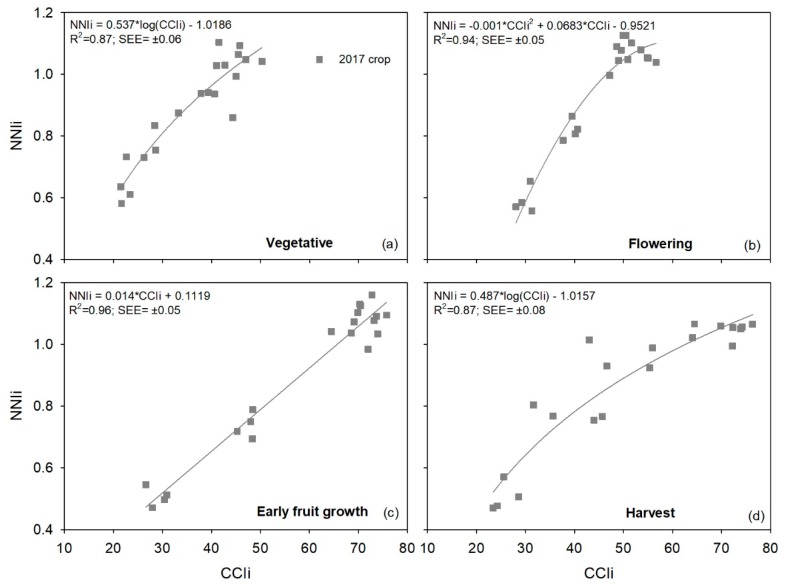
Relationships between integrated chlorophyll content index (*CCIi*) values and the integrated crop nitrogen nutrition index (*NNIi*) for each phenological stage in a sweet pepper (*Capsicum annuum*) crop in 2017. The crop was subjected to five different N treatments with four repetitions. Results of regression are in Table 8.

Relationships between *CCIi* and *NNIi* values for each phenological stage of the 2017 crop had *R*^2^ values of 0.87–0.96 (Table 8). The lowest *R*^2^ value was observed in both the vegetative and harvest stages, and the highest value in the early fruit growth stage. The average *R*^2^ value for all four phenological stages was 0.91 (Table 8). There was no indication of saturation of *CCIi* values at higher *NNI* values in any of the four phenological stages (Figure 5).

### 3.4. Sufficiency Values of Chlorophyll Meters Measurements

The sufficiency values for each phenological stage of each chlorophyll meter for maximum crop growth were derived, from the relationship between the integrated chlorophyll meter measurements of a given phenological stage and integrated *NNI* value of that phenological stage. Sufficiency values for the three chlorophyll meters for each of the four phenological stages in each of the three crops, and when the three crops were considered together are presented in Table 9.

SPAD sufficiency values in the vegetative stage were lower than for the other three phenological stages in each of the three crops. The average value for the vegetative stage of the three crops considered together was 49.7 ± 2.3 SPAD units. Sufficiency values for the flowering stage were intermediate between the vegetative and the early fruit growth and harvest stages, which were similar. The average sufficiency value for the flowering stage of the three crops considered together was 56.6 ± 4.6 SPAD units. SPAD sufficiency values for the early fruit growth and harvest stages were similar in the 2016 and 2017 crops, the average value for both stages for both crops was 61.4 ± 0.6 SPAD units. In the 2014 crop, sufficiency values of these two phenological stages were slightly higher, with the average value for both stages being 66.5 ± 2.4 SPAD units. The SPAD sufficiency values for the early fruit growth and harvest phenological stages of the three crops, considered together, were 62.7 ± 2.3 and 65.2 ± 6.3 SPAD units, respectively (Table 9). Averaged across all four phenological stages and the three crops, the single SPAD sufficiency value for the entire crop was 58.6 ± 3.5 SPAD units.

Sufficiency atLEAF values were lowest in the vegetative stage, intermediate in the flowering stage, and highest in the early fruit growth and harvest stages, for both crops (Table 9). Sufficiency atLEAF values for each phenological phase averaged for the two crops considered together ranged between 51.6 ± 1.9 atLEAF units (vegetative stage) and 58.1 ± 1.5 atLEAF units (early fruit growth stage) (Table 9). Averaged across all four phenological stages and the two crops, the atLEAF sufficiency value for the entire crop was 54.9 ± 0.8 atLEAF units.

Sufficiency values of CCI, measured with the MC-100 meter in the 2017 crop, were lowest in the vegetative stage (42.9 ± 4.9), intermediate in the flowering stage (46.5 ± 3.4), and highest in the early fruit growth and harvest stages (average value for the two stages of 64.3 ± 1.5) (Table 9). Averaged across all four phenological stages, the CCI sufficiency value for the entire crop was 54.5 ± 5.7.

The relative differences in sufficiency values between phenological stages were notably larger for CCI than for SPAD and atLEAF. In the 2017 crop, which was the only crop in which all three chlorophyll meters were used, relative differences in the sufficiency values between the flowering and vegetative stages were 5.4% for SPAD, 2.8% for atLEAF, and 7.7% for CCI. The respective relative differences in the sufficiency values between the early fruit growth and flowering stages were 10.9% (SPAD), 7.0% (atLEAF), and 29.3% (CCI).

## 4. Discussion

Integrated measurements of the three chlorophyll meters (SPAD-502, atLEAF+, and MC-100) were very strongly related to integrated *NNI* for: (a) each of the four phenological stages (vegetative, flowering, early fruit growth, and harvest) of each pepper crop, (b) each crop considered in its entirety, (c) individual phenological stage, using composite data for all crops in which measurements were made, and (d) single values for the entirety of the crop, for the crops in which measurements were made. These results demonstrate that the three chlorophyll meters provided good estimations of the crop N status of sweet pepper. This is in agreement with studies that reported strong relationships between chlorophyll meter measurements and crop N status, in various horticultural [4,17,32] and cereal crops [33,34,35].

Considering the four individual phenological stages, the strongest relationships between the three integrated chlorophyll meter measurements and *NNIi* were obtained in the flowering and early fruit growth stages, which occurred in the middle of the crops, for individual crops, and for when data was combined from multiple crops. Similarly, the strongest relationship between chlorophyll meter measurements and leaf N concentration occurred in the middle of the growing season in potatoes [3]. In the present study, there were also strong relationships at the beginning (in the vegetative stage) and at the end of the crop (in the harvest stage), but with slightly lower R^2^ values than in the flowering and early fruit growth stages. The strong relationship in the vegetative stage of sweet pepper, in this study, contrasts with the results for cucumber in a previous study, where there was a weak relationship between SPAD measurements and *NNI* in the vegetative stage [17], which was attributed to limited differentiation of the N treatments at the beginning of that crop [17]. In the current study, the high R^2^ values between the integrated chlorophyll meters measurements and *NNIi* in each of the four phenological stages, regardless of year and chlorophyll meter, demonstrated the robust ability of chlorophyll meter measurements to be used as indicators of the crop N status of sweet pepper.

For the three chlorophyll meters, there was no evidence of saturation when relating measurements to *NNIi* in any of the four phenological stages and in the different crops. Regression analysis showed that *SPADi*, *atLEAFi,* and *CCIi* values increased when *NNIi* values exceeded the optimal value for the crop growth of one. Saturation of SPAD-502 and atLEAF+ measurements at high chlorophyll contents, which are associated with high crop N contents has been often reported [11,33,36]. However, saturation of chlorophyll meter measurements does not always occur at higher crop N contents [3,4], as it depends on whether leaf chlorophyll contents are sufficiently high to cause saturation [11]. None of the three chlorophyll meters evaluated were able to differentiate between the N4 and N5 treatments. This was not due to a saturation response of the chlorophyll meters, but rather was due to the similar crop N status of these two treatments, as indicated by the very similar *NNIi* values. The *NNIi* values of treatments N4 and N5 were not significantly different for any of the three crops.

Regarding the calculation of sufficiency values of the SPAD-502 meter, there were only small differences in sufficiency values for each phenological stage between the three different crops. Similarly, there were only small differences between sufficiency values for individual crops and the corresponding sufficiency value for the combined crop data set, for a given phenological stage. These data indicate that the sufficiency values determined for each phenological stage were very consistent between the three different years, and that SPAD sufficiency values obtained with the combined dataset are representative of the three crops.

The relative constancy of SPAD sufficiency values over time can be assessed by comparing the sufficiency values of the different phenological stages, using the combined data of the three crops. The relative difference between the sufficiency values of the vegetative and flowering stages was 12.2%, between flowering and early fruit growth was 9.7%, and between the early fruit growth and harvest stages was 3.8%. The differences between the sufficiency values for successive stages diminished as the crop grew. This was attributed to the temporal dynamics of chlorophyll meter values (Figure 2) because SPAD measurements increased in the early part of the crops and reached relatively stable values midway through the crops. There was a large difference in SPAD sufficiency values between the harvest stage (last phenological stage of the crop) and the vegetative stage (first phenological stage of the crop) of 15.5 SPAD units, the relative difference being 23.4%. This large difference during the crop suggests that a single SPAD sufficiency value cannot be used for a whole sweet pepper crop. In contrast, single SPAD sufficiency values for a whole crop have been proposed for cucumber [17,22] and grapevine [37].

The SPAD sufficiency values derived for sweet pepper, in the present study, are generally higher than reported for other horticultural crops; the highest sufficiency value obtained in the present work was 64.0 ± 1.3 SPAD units. In indeterminate tomato, the average sufficiency value for the complete crop cycle was 54.2 SPAD units [21]. In cucumber, sufficiency values have been recommended for the whole crop of 45.2 SPAD units [17] and 44.9 SPAD units [22]. In potato, a whole crop sufficiency value of 38.2 SPAD units was recommended [38]. The appreciably higher sufficiency values for the SPAD-502 meter for sweet pepper in the present work can be explained by the very high leaf chlorophyll content of sweet pepper [11]. In a study with 22 common crop species, sweet pepper had the highest leaf chlorophyll concentration, which was double that of maize [39].

The performance of the atLEAF+ and MC-100 meters was similar to the SPAD-502 meter in terms of sufficiency values. With both the atLEAF+ and MC-100 meters, the lowest sufficiency values were in the vegetative stage, and the highest in the early fruit growth and harvest stages. As with the SPAD, these temporal variations were associated with the dynamics of chlorophyll meter measurements throughout the crop, which initially increased and then were relatively constant in the second half of the crop. For the atLEAF+ meter, there were only small differences in the sufficiency value, for each of the four phenological stages, between the 2016 and 2017 crops. This indicates that the atLEAF sufficiency values determined were consistent between the two crops. It also demonstrates that the sufficiency values calculated using the combined data set of the two crops are representative of both crops. For the MC-100 meter, this is one of the first studies to provide sufficiency values of CCI. With this chlorophyll meter in the current study, there was only one crop; so, it was not possible to assess the consistency of sufficiency values between crops. The relative differences in sufficiency values between each of the four phenological stages, for the atLEAF+ sensor and the MC-100 meter, were calculated to assess the consistency of sufficiency values for each chlorophyll meter over time. The atLEAF+ meter had the narrowest range in sufficiency values, with the difference between the early fruit growth stage (maximum sufficiency value) and the vegetative stage (minimum sufficiency value) being 11.2%. The largest range of sufficiency values was with the MC-100 meter, where the relative difference between the maximum sufficiency value (early fruit growth stage) and the minimum sufficiency value (vegetative stage) was 34.0%.

Following the evaluation and the derivation of sufficiency values, chlorophyll meters could be used to frequently assess the crop N status of fertigated pepper crops that frequently receive N by regular drip irrigation. In greenhouses in SE Spain, N and other nutrients are applied every one to four days in each irrigation. Sufficiency values are required for practical real time monitoring of crop N status, using chlorophyll meters. Frequent effective monitoring of crop N status will enable rapid correction of crop N status by adjusting mineral N fertilizer application when chlorophyll meter measurements deviate from sufficiency values [4], thereby ensuring optimal N nutrition. This will also reduce excessive “insurance” N applications that are applied to avoid the risk of N deficiency. In crops grown with fertigation systems, where N is applied in every irrigation, adjustment in N fertilization can be made very soon after such deviations are detected [5]. The results obtained in this study may be applied to sweet pepper crops grown in greenhouses; for sweet pepper crops grown outdoors, further research is required to validate these sufficiency values.

Overall, the results of this study show the potential of chlorophyll meters for monitoring crop N status and to assist with N fertilizer management of sweet pepper. The strong relationship between integrated chlorophyll meter measurements and *NNIi* for each phenological stage of each crop, when considered separately and as a combined dataset from different crops, demonstrated the consistency and robustness of chlorophyll meter measurements as indicators of crop N status. The sufficiency values calculated for chlorophyll meter measurements in each phenological stage and their consistency throughout crops showed the potential for the sufficiency values to be used in commercial farming to achieve improved N management of sweet pepper crops.

## Figures and Tables

**Table 1:** Mineral N (NO_3_^−^–N + NH_4_^+^–N) concentration in the nutrient solution and mineral N amount applied in fertigation in the three sweet pepper crops.

	2014		2016		2017	
N Treatment	N Concentration (mmol L^−1^)	N Amount (kg ha^−1^)	N Concentration (mmol L^−1^)	N Amount (kg ha^−1^)	N Concentration (mmol L^−1^)	N Amount (kg ha^−1^)
N1—Very deficient	2.4	64	2.0	88	2.0	86
N2—Deficient	6.2	189	5.3	302	5.7	304
N3—Conventional	12.6	516	9.7	561	9.7	519
N4—Excessive	16.1	804	13.5	1052	13.1	870
N5—Very excessive	20.0	990	17.7	1320	16.7	1198

**Table 2:** Integrated nitrogen nutrition index (*NNIi*) values for each N treatment within each phenological stage in each of the three sweet pepper (*Capsicum annuum*) crops. Different lower-case letters (a–d) show significant differences between N treatments within each phenological stage and crop, after the least significant difference (LSD) post-hoc test of ANOVA. *p*-value < 0.001. Values are means (n = 4) ± standard error (SE). Each crop was subjected to five different N treatments with four repetitions.

Phenological Stage	Treatment	2014 Crop	2016 Crop	2017 Crop
Vegetative	N1	0.82 ± 0.02^a^	0.65 ± 0.04^a^	0.64 ± 0.03^a^
	N2	0.94 ± 0.02^b^	0.79 ± 0.04^b^	0.80 ± 0.03^b^
	N3	1.06 ± 0.02^c^	0.88 ± 0.02^c^	0.92 ± 0.02^c^
	N4	1.12 ± 0.02^d^	0.98 ± 0.01^d^	1.05 ± 0.02^d^
	N5	1.12 ± 0.01^d^	1.01 ± 0.02^d^	1.05 ± 0.02^d^
Flowering	N1	0.59 ± 0.01^a^	0.62 ± 0.04^a^	0.59 ± 0.02^a^
	N2	0.80 ± 0.02^b^	0.89 ± 0.03^b^	0.82 ± 0.02^b^
	N3	1.11 ± 0.02^c^	0.92 ± 0.03^b^	1.05 ± 0.03^c^
	N4	1.13 ± 0.01^c^	1.06 ± 0.03^c^	1.07 ± 0.02^c^
	N5	1.11 ± 0.02^c^	1.10 ± 0.02^c^	1.08 ± 0.02^c^
Early fruit growth	N1	0.58 ± 0.03^a^	0.62 ± 0.05^a^	0.51 ± 0.02^a^
	N2	0.81 ± 0.02^b^	0.85 ± 0.01^b^	0.74 ± 0.02^b^
	N3	1.03 ± 0.03^c^	0.99 ± 0.01^c^	1.02 ± 0.01^c^
	N4	1.09 ± 0.01^cd^	1.11 ± 0.03^d^	1.09 ± 0.01^d^
	N5	1.10 ± 0.00^d^	1.11 ± 0.03^d^	1.12 ± 0.02^d^
Harvest	N1	0.56 ± 0.02^a^	0.74 ± 0.01^a^	0.51 ± 0.02^a^
	N2	0.77 ± 0.01^b^	0.93 ± 0.01^b^	0.77 ± 0.01^b^
	N3	0.99 ± 0.00^c^	1.01 ± 0.02^c^	0.96 ± 0.02^c^
	N4	1.02 ± 0.01^cd^	1.07 ± 0.02^d^	1.05 ± 0.01^d^
	N5	1.03 ± 0.00^d^	1.03 ± 0.02^cd^	1.05 ± 0.02^d^

**Table 3:** Integrated SPAD (*SPADi*) values for each N treatment within each phenological stage in each of the three sweet pepper (*Capsicum annuum*) crops. Different lower-case letters (a–d) show significant differences between N treatments within each phenological stage and crop, after LSD post-hoc test of ANOVA. *p*-value < 0.001. Values are means (n = 4) ± standard error (SE). Each crop was subjected to five different N treatments with four repetitions.

Phenological Stage	Treatment	2014 Crop	2016 Crop	2017 Crop
Vegetative	N1	45.0 ± 0.1^a^	42.5 ± 0.2^a^	40.8 ± 0.2^a^
	N2	47.3 ± 0.3^b^	46.4 ± 0.8^b^	44.7 ± 0.9^b^
	N3	50.2 ± 0.4^c^	47.3 ± 0.7^bc^	50.8 ± 0.7^c^
	N4	51.2 ± 0.6^cd^	48.6 ± 0.6^bc^	52.0 ± 0.4^c^
	N5	52.0 ± 0.5^d^	49.5 ± 1.1^c^	51.8 ± 0.7^c^
Flowering	N1	47.7 ± 0.7^a^	51.1 ± 0.7^a^	45.8 ± 0.5^a^
	N2	52.5 ± 0.1^b^	57.6 ± 0.4^b^	50.9 ± 0.4^b^
	N3	57.9 ± 0.5^c^	59.5 ± 0.5^c^	55.2 ± 0.5^c^
	N4	58.8 ± 0.2^cd^	61.4 ± 0.4^d^	55.8 ± 0.6^c^
	N5	59.6 ± 039^d^	61.3 ± 0.4^d^	55.8 ± 0.3^c^
Early fruit growth	N1	49.8 ± 1.0^a^	55.9 ± 0.6^a^	45.7 ± 0.5^a^
	N2	59.1 ± 0.3^b^	59.4 ± 0.4^b^	54.7 ± 0.2^b^
	N3	64.5 ± 0.4^c^	62.5 ± 0.7^c^	61.8 ± 0.7^c^
	N4	65.5 ± 0.4^cd^	64.5 ± 0.2^d^	62.4 ± 0.5^c^
	N5	67.0 ± 0.4^d^	65.2 ± 0.3^d^	62.7 ± 0.4^c^
Harvest	N1	52.7 ± 1.2^a^	54.9 ± 0.7^a^	43.2 ± 0.8^a^
	N2	63.2 ± 0.3^b^	59.7 ± 0.3^b^	51.2 ± 1.9^b^
	N3	67.4 ± 0.3^c^	62.4 ± 0.2^c^	55.9 ± 1.6^c^
	N4	69.3 ± 0.2^d^	63.0 ± 0.4^cd^	62.3 ± 0.6^d^
	N5	70.0 ± 0.3^d^	63.7 ± 0.4^d^	62.3 ± 1.0^d^

**Table 4:** Integrated atLEAF (*atLEAFi*) values for each N treatment within each phenological stage in the two sweet pepper (*Capsicum annuum*) crops. Different lower-case letters (a–d) show significant differences between treatments within each phenological stage and crop, after LSD post-hoc test of ANOVA. *p*-value < 0.001. Values are means (n = 4) ± standard error (SE). Each crop was subjected to five different N treatments with four repetitions.

Phenological Stage	Treatment	2016 Crop	2017 Crop
Vegetative	N1	45.1 ± 0.6^a^	42.7 ± 0.3^a^
	N2	46.8 ± 0.6^ab^	46.1 ± 0.6^b^
	N3	48.4 ± 0.4^bc^	51.3 ± 0.4^c^
	N4	50.1 ± 0.7^cd^	51.6 ± 0.4^c^
	N5	51.0 ± 1.1^d^	52.4 ± 0.6^c^
Flowering	N1	48.2 ± 0.6^a^	46.7 ± 0.4^a^
	N2	53.2 ± 0.3^b^	50.2 ± 0.4^b^
	N3	54.3 ± 0.2^b^	53.5 ± 0.3^c^
	N4	55.8 ± 0.3^c^	54.5 ± 0.3^d^
	N5	55.9 ± 0.2^c^	54.5 ± 0.2^d^
Early fruit growth	N1	53.8 ± 0.6^a^	46.5 ± 0.7^a^
	N2	56.7 ± 0.6^b^	53.0 ± 0.4^b^
	N3	58.8 ± 0.6^c^	58.1 ± 0.4^c^
	N4	59.4 ± 0.1^cd^	58.0 ± 0.6^c^
	N5	60.3 ± 0.2^d^	58.2 ± 0.2^c^
Harvest	N1	53.4 ± 0.3^a^	43.1 ± 0.8^a^
	N2	56.3 ± 0.4^b^	49.2 ± 0.8^b^
	N3	57.2 ± 0.2^bc^	52.2 ± 0.8^c^
	N4	57.3 ± 0.2^c^	55.9 ± 0.4^d^
	N5	58.0 ± 0.2^c^	55.6 ± 0.4^d^

**Table 5:** Integrated CCI (*CCIi*) values for each N treatment within each phenological stage in the 2017 sweet pepper (*Capsicum annuum*) crop. Different lower-case letters (a–d) show significant differences between N treatments within each phenological stage, after LSD post-hoc test of ANOVA. *p*-value < 0.001. Values are means (n = 4) ± standard error (SE). The crop was subjected to five different N treatments with four repetitions.

Phenological Stage	Treatment	*CCIi*
Vegetative	N1	22.3 ± 0.5^a^
	N2	29.1 ± 1.5^b^
	N3	40.5 ± 1.4^c^
	N4	44.8 ± 1.2^c^
	N5	45.0 ± 2.0^c^
Flowering	N1	29.9 ± 0.8^a^
	N2	39.5 ± 0.7^b^
	N3	49.2 ± 0.8^c^
	N4	52.9 ± 1.8^d^
	N5	52.2 ± 1.3^cd^
Early fruit growth	N1	29.0 ± 1.0^a^
	N2	47.5 ± 0.8^b^
	N3	69.7 ± 2.1^c^
	N4	71.6 ± 1.1^c^
	N5	72.2 ± 1.4^c^
Harvest	N1	25.5 ± 1.1^a^
	N2	39.2 ± 3.4^b^
	N3	50.2 ± 3.2^c^
	N4	71.1 ± 2.4^d^
	N5	70.7 ± 2.5^d^

**Table 6:** Coefficients of determination (R^2^) of regressions between integrated SPAD (*SPADi*) values and integrated nitrogen nutrition index (*NNIi*) for each phenological stage in each of the three sweet pepper (*Capsicum annuum*) crops independently, and for the three crops together. Each crop was subjected to five different N treatments with four repetitions. According to the Akaike information criterion, the best-fit regression model (exponential, linear, power, quadratic, and natural logarithm) is shown. Also, the fitted equation and standard error of the estimate (SEE) are presented. All regressions were highly significant at *p*-value <0.001. N is the number of data points of regressions.

Crop	Phenological Stage	Regression	Equation	*R* ^2^	SEE (±*NNIi*)	N
2014	Vegetative	Quadratic	*NNIi* = −0.004∙*SPADi^2^* + 0.4324∙*SPADi* − 10.5	0.89	0.04	20
	Flowering	Linear	*NNIi* = 0.047 *SPADi* − 1.6315	0.95	0.05	20
	Early fruit growth	Exponential	*NNIi* = 0.087e^0.038∙*SPADi*^	0.92	0.05	20
	Harvest	Exponential	*NNIi* = 0.082e^0.0362∙*SPADi*^	0.97	0.04	20
2016	Vegetative	Natural Logarithm	*NNIi* = 2.127∙log(*SPADi*) − 7.319	0.80	0.07	20
	Flowering	Linear	*NNIi* = 0.043∙*SPADi* − 1.5753	0.88	0.07	20
	Early fruit growth	Natural Logarithm	*NNIi* = 3.079∙log(*SPADi*) − 11.741	0.88	0.07	20
	Harvest	Natural Logarithm	*NNIi* = 1.981∙log(*SPADi*) − 7.1744	0.86	0.05	20
2017	Vegetative	Natural Logarithm	*NNIi* = 1.517∙log(*SPADi*) − 4.9752	0.86	0.07	20
	Flowering	Natural Logarithm	*NNIi* = 2.379∙log(*SPADi*) − 8.5002	0.92	0.06	20
	Early fruit growth	Exponential	*NNIi* = 0.064∙e^0.0453∙*SPADi*^	0.96	0.06	20
	Harvest	Natural Logarithm	*NNIi* = 1.349∙log(*SPADi*) − 4.523	0.87	0.08	20
2014 + 2016 + 2017	Vegetative	Quadratic	*NNIi* = −0.002∙*SPADi^2^* + 0.1935∙*SPADi* − 4.57	0.82	0.07	60
	Flowering	Quadratic	*NNIi* = −0.002∙*SPADi^2^* + 0.2031∙*SPADi* − 5.5115	0.73	0.11	60
	Early fruit growth	Power	*NNIi* = 0.00007∙*SPADi*^2.3116^	0.85	0.08	60
	Harvest	Natural Logarithm	*NNIi* = 1.125∙log(*SPADi*) − 3.6978	0.64	0.11	60

**Table 7:** Coefficients of determination (R^2^) of regressions between integrated atLEAF (*atLEAFi*) values and integrated nitrogen nutrition index (*NNIi*) for each phenological stage in each of the two sweet pepper (*Capsicum annuum*) crops independently and for the two crops together. Each crop was subjected to five different N treatments with four repetitions. According to the Akaike information criterion, the best-fit regression model (exponential, linear, power, quadratic, and natural logarithm) is shown. Also, it is presented the fitted equation and standard error of the estimate (SEE). All regressions were highly significant, with *p*-value < 0.001. N is the number of data points of regression.

Crop	Phenological Stage	Model	Equation	*R* ^2^	SEE (±*NNIi*)	N
2016	Vegetative	Natural Logarithm	*NNIi* = 2.333∙log(*atLEAFi*) − 8.1826	0.74	0.08	20
	Flowering	Linear	*NNIi* = 0.057∙*atLEAFi* − 2.1224	0.85	0.07	20
	Early fruit growth	Natural Logarithm	*NNIi* = 4.042∙log(*atLEAFi*) − 15.458	0.84	0.08	20
	Harvest	Natural Logarithm	*NNIi* = 3.561∙log(*atLEAFi*) − 13.406	0.81	0.06	20
2017	Vegetative	Natural Logarithm	*NNIi* = 1.841∙log(*atLEAFi*) − 6.2617	0.84	0.07	20
	Flowering	Natural Logarithm	*NNIi* = 3.117∙log(*atLEAFi*) – 11*.38	0.94	0.05	20
	Early fruit growth	Exponential	*NNIi* = 0.026∙e^0.0637∙*atLEAFi*^	0.93	0.07	20
	Harvest	Natural Logarithm	*NNIi* = 1.996∙log(*atLEAFi*) − 6.9817	0.90	0.07	20
2016 + 2017	Vegetative	Natural Logarithm	*NNIi* = 1.988∙log(*atLEAFi*) − 6.8381	0.79	0.07	40
	Flowering	Natural Logarithm	*NNIi* = 2.828∙log(*atLEAFi*) − 10.284	0.83	0.08	40
	Early fruit growth	Exponential	*NNIi* = 0.027∙e^0.0617∙*atLEAFi*^	0.82	0.10	40
	Harvest	Natural Logarithm	*NNIi* = 1.724∙log(*atLEAFi*) − 5.9541	0.77	0.09	40

**Table 8:** Coefficients of determination (*R*^2^) of regressions between integrated chlorophyll content index (*CCIi*) values and the integrated nitrogen nutrition index (*NNIi*) for each phenological stage in a sweet pepper (*Capsicum annuum*) in 2017. The crop was subjected to five different N treatments with four repetitions. According to the Akaike information criterion, the best-fit regression model (exponential, linear, power, quadratic, and natural logarithm) is shown. Also, it is presented the fitted equation and standard error of the estimate (SEE). All regressions were highly significant, with *p*-value < 0.001. N is the number of data points of regression.

Phenological Stage	Model	Equation	*R* ^2^	SEE (±*NNIi*)	N
Vegetative	Natural Logarithm	*NNIi* = 0.537∙log(*CCIi*) − 1.0186	0.87	0.06	20
Flowering	Quadratic	*NNIi* = −0.001∙*CCIi^2^* + 0.0683∙*CCIi* − 0.9521	0.94	0.05	20
Early fruit growth	Linear	*NNIi* = 0.014∙*CCIi* + 0.1119	0.96	0.05	20
Harvest	Natural Logarithm	*NNIi* = 0.487∙log(*CCIi*) − 1.0157	0.87	0.08	20

**Table 9:** Sufficiency values of SPAD, atLEAF, and CCI, in each of the four phenological stages, for individual sweet pepper (*Capsicum annuum*) crops and for all years combined. Values are means ± standard error (SE).

Crop	Phenological Stage	SPAD	atLEAF	CCI
2014	Vegetative	48.1 ± 1.0	/	/
	Flowering	56.4 ± 1.1		
	Early fruit growth	64.1 ± 1.4		
	Harvest	68.8 ± 0.1		
2016	Vegetative	49.9 ± 1.5	51.2 ± 1.7	/
	Flowering	60.1 ± 1.5	54.9 ± 1.3	
	Early fruit growth	62.7 ± 1.4	58.7 ± 1.2	
	Harvest	61.9 ± 1.5	57.1 ± 0.9	
2017	Vegetative	51.3 ± 2.2	51.6 ± 1.9	42.9 ± 4.9
	Flowering	54.2 ± 1.3	53.1 ± 0.8	46.5 ± 3.4
	Early fruit growth	60.8 ± 1.2	57.1 ± 1.1	65.7 ± 4.0
	Harvest	60.1 ± 3.5	54.5 ± 1.9	62.8 ± 10.1
All years	Vegetative	49.7 ± 2.3	51.6 ± 1.9	/
	Flowering	56.6 ± 4.6	54.1 ± 1.5	
	Early fruit growth	62.7 ± 2.3	58.1 ± 1.5	
	Harvest	65.2 ± 6.3	56.5 ± 2.9	
